# Factors affecting the performance of periodontal specialty in secondary oral health care in Brazil

**DOI:** 10.1371/journal.pone.0287361

**Published:** 2023-10-12

**Authors:** Vinícius de Moraes Simião, Estéfany Figueiredo Gonzalez, Livia Fernandes Probst, Rafaela da Silveira Pinto, Alessandro Diogo De-Carli

**Affiliations:** 1 Postgraduate Program in Family Health, Universidade Federal de Mato Grosso do Sul, Campo Grande, Mato Grosso do Sul, Brazil; 2 Multiprofessional Residency Program in Family Health SESAU/Fiocruz, Campo Grande, Mato Grosso do Sul, Brazil; 3 Health Technology Assessment Unit, Hospital Alemão Oswaldo Cruz, São Paulo, São Paulo, Brazil; 4 Department of Social and Preventive Dentistry, Universidade Federal de Minas Gerais, Belo Horizonte, Minas Gerais, Brazil; 5 Faculty of Dentistry, Universidade Federal de Mato Grosso do Sul, Campo Grande, Mato Grosso do Sul, Brazil; Federal University of Juiz de Fora, BRAZIL

## Abstract

**Objective:**

The aim of this study was to investigate, at a national level, which individual factors of the work process/infrastructure are associated with the achievement of goals in the periodontics specialty in Brazilian Dental Specialty Centers (BDSC).

**Methods:**

This was a quantitative, analytical, cross-sectional study. Secondary data from DATASUS and the external evaluation of the second cycle of the BDSC Access and Quality Improvement Program were used. Variable description was carried out in the first stage, and then the bivariate Poisson regression was performed to verify possible associations between the variables and the outcome (achievement of goals in Periodontics in the BDSC). In this analysis, the covariates that were associated with the outcome at the p <0.20 significance level were included in the next step of the analysis. Multivariate Poisson regression with a robust estimator was then performed with those that met the above criterion. The variables that showed a p value < 0.05 were considered in the final model.

**Results:**

The outcome was achieved in more than seven months of the year (mean 7.03 months, SD 4.20). Most BDSC monitored the established goals (93.2%), had referral as the only way of access (61.7%), had only municipal coverage (68.4%), carried out planning and periodic evaluation of actions (89.2%). A minority has quotas of procedures by Oral Health teams (OHTs) in Primary Health Care (PHC) (18.8%). The presence of a specialist in periodontics was (on average) 1.16 per BDSC and the sum of the workload of dentists working in this specialty was 31.1 hours (SD = 23.9).

**Conclusion:**

It was concluded that the individual factors of the work process/infrastructure associated with the achievement of goals in periodontics in Brazilian BDSC are: monitoring of established goals, BDSC scope and number of professionals working in the specialty.

## Introduction

With the implementation of the National Oral Health Policy (PNSB, *Política Nacional de Saúde Bucal*) in 2004, by the Ministry of Health (MoH), oral health care in the Brazilian Unified Health System (SUS, *Sistema Único de Saúde*), historically characterized by difficult access and limited to mutilating techniques, started to include the promotion, prevention and recovery of oral health of the Brazilian population. Therefore, the reorganization and qualification of the service provided by the SUS was crucial [1,2].

From this perspective, the PNSB promoted the expansion of the coverage provided by the oral health teams (OHTs) in the Family Health Strategy (FHS) to reorganize access to oral health in Primary Health Care (PHC) and implemented the Brazilian Dental Specialty Centers (BDSC), aiming to expand access to Secondary Oral Health Care (SOHC) [1,2]. The BDSC are health care establishments listed in the National Register of Health Establishments (CNES, *Cadastro Nacional de Estabelecimentos de Saúde*), which are obliged to offer basic oral health services in the Oral Diagnosis, Advanced Periodontics, Minor Oral Surgery, Endodontics and Dental Care specialties to patients with special needs. The granting of funding for the BDSC maintenance is linked to the achievement of goals by the specialty [1].

The BDSC are classified according to their composition regarding the number of dental chairs in the establishment. Therefore, Modality I, II and III of the BDSC consist of three, four to six, and more than seven dental chairs, respectively. These establishments operate for 40 hours a week and the number of professionals working there varies according to the modality, with the achievement of goals being monitored by the MoH [3].

However, in addition to offering greater access to SOHC, it was necessary to invest in evaluation processes at this level of care, aiming to attain better quality of the services offered by the BDSC. For this purpose, the MoH implemented the BDSC component in the National Access and Quality Improvement Program (PMAQ, *Programa Nacional de Melhoria do Acesso e da Qualidade*) through Ordinance GM/MoH N. 261, of February 21, 2013, having its rules revised by Ordinance GM/MoH N. 1,599, of September 30, 2015 [3]. The PMAQ-CEO, until 2018, corresponded to the federal initiative for the evaluation and monitoring of the SOHC in Brazil.

This study aims to assess the achievement of the goals in the periodontics specialty of Brazilian BDSC. In this specialty, the goal to be achieved varies according to the type of BDSC, comprising 60 procedures for BDSC type I, 90 for type II and 150 for type III [4], which were verified by the PMAQ-CEO evaluation process in its second cycle, in 2018 [5].

The epidemiological picture of periodontal conditions in Brazil is still considered inconclusive [6]. However, if one considers the CDC/AAP (Centers for Disease Control/ American Academy of Periodontology) criteria, the prevalence of periodontal disease in Brazil is still higher than in developed countries [6]. In the United States, the prevalence of severe periodontitis ranges from 6.7 to 11.7%, while in Brazil it ranges from 34.4% to 63.8% in individuals aged 35 years or older [6].

The need to analyze compliance with periodontics goals in the BDSC is justified, as there are no studies in this area with this specific approach, making it an unprecedented analysis. Moreover, it is necessary to consider the critical role of BDSC in relation to the attention/control of periodontal disease, considering that, in terms of public policy, it corresponds to the only access for most Brazilians to periodontal care at a specialized level, via SUS.

The aim of this study is to investigate, at a national level, which individual factors are associated with the achievement of goals in the periodontics specialty in Brazilian BDSC.

## Method

### Ethical aspects

The microdata used in this study were obtained from National Information Systems with public and unrestricted access.

The PMAQ-CEO was approved by the Research Ethics Committee (CEP) of the Federal University of Pernambuco (UFPE), under CAAE number: 23458213.0.0000.5208, complying with the requirements of Resolution n. 466/12 of the National Health Council.

### Study design and context

This is an analytical cross-sectional study using secondary data and reported in accordance with the Strengthening the Reporting of Observational Studies in Epidemiology (STROBE) statement [7]. The study uses data from the external evaluation of the 2^nd^ cycle of the National Program to Improve Access and Quality of Dental Specialty Centers (PMAQ-CEO), carried out in 2018, in Brazil [3]. Microdata related to modules I and II of the PMAQ-CEO were used, which evaluated the structure and work process in these establishments, respectively.

### Study universe and sample

The universe of the study comprises the municipalities in which the BDSC adhered to the PMAQ and who answered the external evaluation questionnaire. Of the BDSC implemented in the Brazilian territory in 2018, 1097 answered the external evaluation questionnaire. Of these, 104 were excluded for showing zero production in all months of the year, 15 for not having identified the type of BDSC and 05 for not providing individual data in the database of the PMAQ-CEO 2^nd^ cycle. Therefore, the final sample consisted of 973 BDSC from 809 municipalities in Brazil. The authors did not had access to information that could identify individual participants during or after data collection.

### Analyzed variables

The variables used in the study are described in Chart 1.

**Chart 1:** Variables (outcome and independent) used in the study.

Variable	Description of the variable	Categories	Data source
***Outcome***			
Goal in the periodontics specialty	Number of months in which the goal was achieved in the periodontics specialty	Achieved the goal. Did not achieve the goal.	DATA-SUS
***Independent variables of the 1***^***st***^ ***level*: *related to BDSC (institutional level)***.			
Type of access	What type of demand determines access to Periodontics at the BDSC?	Referral only. Mixed (spontaneous and referral)	PMAQ-CEO
Quotas of procedures per oral health team in Primary Health Care	Are there predefined quotas set by the Basic Oral Health team for referring users to the BDSC in the specialty of Periodontics?	Yes No	PMAQ-CEO
Monitoring of established goals	Is monitoring and analysis of established goals carried out for each specialty offered at the BDSC?	Yes No	PMAQ-CEO
Sum of periodontics workload	What is the total weekly workload of the dentists working in the Periodontics specialty?	Description (Number)	PMAQ-CEO
Number of dentists working in periodontics	How many dentists work in the Periodontics specialty?	Description (Number)	PMAQ-CEO
Planning and periodic evaluation of actions	Are the actions carried out in this BDSC the result of periodic planning and evaluation?	Yes No	PMAQ-CEO
BDSC scope	Does this BDSC have only municipal coverage (is it a reference only for this municipality)?	Yes No	PMAQ-CEO
BDSC type	There are three types of BDSC, and each receives an incentive amount for implementation and funding provided by the Ministry of Health and the type of BDSC varies according to the number of dental chairs: Type I (with 3 dental chairs) Type II (with 4 to 6 dental chairs) Type III (with more than 7 dental chairs)	Type I Type II Type III	PMAQ-CEO

BDSC = Brazilian Dental Specialty Centers; PHC = Primary Health Care; PMAQ-CEO = National Access and Quality Improvement Program of Dental Specialty Centers.

### Data organization and statistical analysis

All secondary data were organized in spreadsheets that comprised the researcher’s own database, using the Microsoft Excel program. All statistical analyses were performed using the SPSS software, version 23.0. Variable description was performed in the first stage, and then the bivariate Poisson regression analysis was used to verify possible associations between the independent variables and the outcome. In this analysis, the covariates that were associated with the outcome at the significance level of p-value <0.20 were included in the next step of the analysis. Poisson multivariate regression with a robust estimator was then performed with those variables that met the previous criterion. The variables that reached a p-value <0.05 were considered in the final model.

## Results

Overall, the BDSC reached goals in the periodontics specialty in more than seven months of the year (mean: 7.03 months, SD: 4.20). Most BDSC monitored the established goals (93.2%), had referral as the only way of access (61.7%), had municipal coverage only (68.4%), and carried out planning and periodic evaluation of actions (89.2%). A minority has quotas for OHT procedures in the PHC (18.8%). The presence of a specialist in periodontics was (on average) 1.16 per BDSC and the sum of the workload of dentists working in this specialty was 31.1 hours (SD = 23.9) (Table 1).

**Table 1:** Analysis between independent variables and the Goal in the periodontics specialty.

Description		Crude analysis		Adjusted analysis	
Categorical independent variables	N (%)	PR (95%CI)	p	PR (95%CI)	p
*Type of access*					
Only spontaneous or mixed	364 (38.3)	1		1	
Referral only	586 (61.7)	1.19 (1.13–1.25)	<0.001	1.16 (1.07–1.25)	<0.001
*Quotas of procedures per oral health team in PHC*					
No	757 (81.2)	1		1	
Yes	175 (18.8)	1.14 (1.08–1.21)	<0.001	1.08 (0.99–1.18)	0.064
*Monitoring of established goals*					
No	65 (6.8)	1		1	
Yes	885 (93.2)	1.37 (1.23–1.53)	<0.001	1.26 (1.00–1.58)	0.047
*Type of BDSC*					
Type I	372 (39.2)	1		1	
Type II	446 (46.9)	1.04 (0.99–1.09)	0.166	0.96 (0.89–1.04)	0.344
Type III	132 (13.9)	1.14 (1.06–1.22)	<0.001	0.92 (0.82–1.04)	0.180
*BDSC scope*					
Regional	300 (31.6)	1		1	
Municipal	650 (68.4)	0.37 (0.21–0.65)	<0.001	0.90 (0.83–0.96)	0.003
*Planning and periodic evaluation of actions*					
No	97 (10.2)	1		1	
yes	853 (89.8)	1.19 (1.10–1.30)	<0.001	1.08 (0.93–1.25)	0.333
**Continuous independent variables**	**Mean** **(SD)**	**PR** **(95%CI)**	**p**	**PR** **(95%CI)**	**p**
*Sum of periodontics workload*	31.1 (23.9)	1.00 (1.00–1.00)	<0.001	1.001 (1.000–1.003)	0.049
*Number of dentists working in periodontics*	1.39 (0.81)	1.16 (1.13–1.19)	<0.001	1.11 (1.07–1.16)	<0.001
**Dependent Variable**	**Mean** **(SD)**				
Number of months in which the goal was achieved in the periodontics specialty	7.03 (4.20)				

SD = Standard Deviation; BDSC = Brazilian Dental Specialty Centers; CI = Confidence Interval; PR = Prevalence Ratio; LR = Likelihood Ratio.

Table 1 shows the analysis for the outcome (number of months in the year in which the BDSC reached their goals in the periodontics specialty). Poisson regression showed this outcome was associated with variables related to the individual level of dental specialty centers. The BDSC that monitors their established goals were 1.37 times more likely to achieve these goals than the ones who did not monitor the established goals. In turn, a higher number of dentists who work in periodontics and BDSC with regional coverage were also variables associated with the achievement of goals.

## Discussion

The findings of the present study demonstrate an association between the achievement of goals in the specialty of Periodontics and factors that are proximal to the work process in BDSC. To date, no publications have been identified with the same research object. Therefore, the information disclosed here and the problematization of the results contribute to the advancement of the construction of knowledge in the specific field of SOHC assessment in Brazil, having Periodontics as a guiding axis.

Different individual factors were identified, which are related to the number of months in which goals were met in Periodontics in BDSC. Therefore, the monitoring of the established goals, the BDSC scope and the number of dentists working in the specialty suggest that issues intrinsic to the work process can contribute to a better performance of these establishments.

The monitoring of goals was significantly associated with the tendency to achieve them, since the monitoring and planning of the provided services are of the utmost importance to achieve good results in the quality indicators and standards [3]. In terms of evaluation, this becomes relevant, considering that procedural issues inherent to the daily routine of the services can be used as support to guide the BDSC team regarding the process of negotiating and contracting goals with managers, as well as defining priorities for improving the service quality based on the recognition of the achieved results, whether they are effective or in need of improvement regarding the intervention strategies [3].

As the establishments that are regional referrals were more successful in meeting the goals, it is plausible that, in these BDSC, as they are located in referral municipalities in the health regions, there may be better infrastructure, organization of patient flow and demand, as well as of the Oral Health Care Network. These factors may contribute to better results in relation to the outcome, when compared to BDSC of municipal scope.

With regard to human resources, the number of dentists who work in the specialty of periodontics in BDSC showed to be an important factor for the achievement of goals. The results show that, on average, there is more than one periodontist per BDSC, which may be an assumption for the high number of procedures performed in this specialty [8].

The data presented herein are relevant because, in addition to being unprecedented, they can be used as guidelines for the planning and implementation of specific actions in the field of Periodontics, considering that a large part of the Brazilian population depends on public health services, especially in secondary oral health care [9]. In this context, this becomes even more important, as it is known that data related to the prevalence of periodontal disease in Brazil are unequal [10]. This can be justified by the characteristics of the country, such as diversity in socioeconomic, demographic, environmental and behavioral factors originating from its large territorial extension [11], which can be impacted by the lack of a care protocol and standardized criteria for characterizing the disease [12] in the SUS.

Regardless of the discrepancy of results, in a study carried out using the Centers for Disease Control/American Academy of Periodontology (CDC/AAP) criteria, considering the limit of clinical attachment loss ≥ 3mm, it is suggested that Brazil has a higher prevalence of major periodontal diseases than that found in more developed countries, ranging from 34.4% to 63.8% in individuals aged 35 or over [6].

Thus, it indicates a challenge for the PNSB in minimizing limitations, especially those related to the work process, so that access to quality and effective services in periodontics can be promoted, impacting the oral health of the population. In this sense, it is necessary to resume the planning of implementation actions aimed at individuals/communities in a situation of greater risk and vulnerability, which is already foreseen in the PNSB [1], but which, since 2016, with the implementation of the fiscal austerity policy, has been suffering cutbacks and devaluation at a national level.

This has become a critical issue, as it is known that, in a country as unequal as Brazil, there is still an association between socioeconomic variables and the achievement of goals in periodontics, where the size of the population and the Municipal Human Development Index (MHDI) are associated with the BDSC performance [13,14], and there is a correlation between contextual and socioeconomic factors, suggesting inequality in the need for periodontal treatment in the Brazilian elderly population [15].

In this study, the number of months in which the goal of procedures was reached in the specialty of periodontics was, on average, more than seven months. The growing trend of BDSC that meet periodontics goals may be related to the investment in oral health actions from 2003 to 2014, when the PNSB provided conditions to expand the supply and the capacity of services provided by this specialty [16].

This study has some limitations. Despite the representativeness of the sample, these data should be generalized with caution, as the BDSC participation in the PMAQ-CEO evaluation process was not mandatory, but voluntary. Secondary data obtained from national information systems with public and unrestricted access offer numerous advantages, such as broad population coverage and low cost for information collection. However, as these data are usually collected in routine health services without a *priori* research purposes, the absence of important information for the analyses of interest can represent significant disadvantages [17].

In turn, the quality of information from databases can be assessed in two dimensions: completeness and accuracy. Completeness refers to the extent to which data are missing from the perspective of the outlined research question. Missing data is unavoidable; however, it is often necessary to understand the extent to which important variables are missing and the possible reasons for their absence. Another important dimension is accuracy. Information from electronic system records, such as procedure codes or numeric values, can sometimes be recorded inaccurately [17].

From an investigative perspective, it is suggested that new studies be carried out, exploring the interface between the Periodontics actions implemented in the PNSB, PHC and the use of the service by the user. Therefore, studies using mixed methods are essential, which have the power to go beyond quantitative generalizations, further analyzing critical issues inherent to the complexity of the health-disease process in the context of the SUS.

This holds significant importance, especially when considering the global scenario, where the prevalence of periodontitis exhibits an inverse correlation with the level of socioeconomic development. While the greatest burden of periodontitis is predominantly observed in individuals aged 55–59, there is a notable increase in its occurrence among younger age groups [18]. In the case of developing countries like Brazil, this becomes critical, considering that the majority of the population (of any age group) relies on the SUS for oral healthcare.

## Conclusion

It was concluded that the factors associated with the achievement of goals in periodontics in BDSC include the monitoring of established goals, the BDSC scope and the number of dentists working in the specialty.
