# Supplementary material for: Factors affecting the performance of periodontal specialty in secondary oral health care in Brazil
Source: PLoS One. 2023 Oct 12;18(10):e0287361. doi: 10.1371/journal.pone.0287361 (PMC10569536; doi:10.1371/journal.pone.0287361)
Supplement: Attachment — Submitted filename: Response to reviewers.docx [file pone.0287361.s001.docx]

Dear Editor,

We thank you for the opportunity to have our manuscript which was originally titled “Factors related to the performance of the periodontal specialty in secondary oral health care in Brazil,” considered for publication at the *Plos One*. The new title of the manuscript is “Factors Affecting the Performance of Periodontal Specialty in Secondary Oral Health Care in Brazil.”

We extend our gratitude to the reviewers for generously devoting their time, efforts, and providing valuable suggestions for improvement. We are pleased to elucidate the reasons behind the authors' decision-making process and their implementation of the suggested changes. These revisions aim to enhance the overall comprehension and significance of this manuscript, thus ensuring its successful publication. The following section describes these actions in detail.

Corrections are highlighted in yellow markers (reviewer #1), green markers (reviewer #2) and green markers (reviewer #3).

Yours sincerely,

The Authors

**Reviewer #1**

Basically, the paper should be understandable just by reading the title, abstract, and tables. In that regard, the following points should be corrected first.

*Our response: We thank the reviewer for the positive feedback. We have revised the paper in response to the constructive and insightful reviewer’s comments.*

The use of the word secondary in the title needs to be clarified. If authors mean secondary analysis, I suggest using something like "Using DATASUS". Please clarify the definition of goal in outcome and suggest whether BDSC is not a more understandable acronym than CEO.

*Our response: We appreciate the suggestion. We have changed the title of the article and made other modifications to ensure greater textual clarity.*

Chart 1

Please write the PHC full name.

sum of periodontics workload is the number of visits?

CEO scope, CEO type. These terms alone do not convey understanding to the reader. The same goes for these expressions, Type 1,2,3.

*Our response: We created a new column with the name 'Description of the variable' and provided descriptions for all variables to ensure the chart 1 is self-explanatory.*

Table 1

It’s undesirable to write ‘study outcome’ as the table title. I recommend to use the name of the specific outcome variables. Make it clear what the goal is, and if it's long, put the meaning in a footnote to the table.

*Our response: We have rewritten the title of Table 1 to address the reviewer's suggestion.*

Typically, the first table shows the results of a univariate analysis of how counfounders differ by dividing the outcome variable into columns before regression analysis. The second table shows the results of the regression, and the third table shows the results stratified by age, gender, or other important variables. In this study, there is only one table with the results, and why is there no demographic information such as age or gender?

*Our response: Dear reviewer, we understand your comment. However, in our article, the 1st level variables represent the team (institutional level), so variables related to gender and age do not apply in this context.*

**Reviewer #2**

Further analysis is needed in the future to analyze and verify the results after implementing the author's recommendations. The literature is needed to highlight the importance of periodontal health in developing countries.

*Our response:* *We appreciate the reviewer's suggestion. We included a paragraph at the end of the Discussion section to emphasize the significance of periodontal health in developing countries.*

**Reviewer #3**

The aim of the study is well written and the objectives defined. The methodology and the statistical analysis are well described and appropriate. As the authors have described in their study, the data maybe used as guidelines for better planning and implementation of specific actions and improving health policies for the public. Improvement in health policies and access to oral care is important to address a prevalence of periodontal disease as high as 34.4%to 63.8%.

*Our response: We thank the reviewer for the positive feedback.* *We included a paragraph at the end of the Discussion section emphasizing the importance of further studies in light of the increasing incidence of periodontal diseases.*
