# Supplementary material for: Factors affecting the performance of periodontal specialty in secondary oral health care in Brazil
Source: PLoS One. 2023 Oct 12;18(10):e0287361. doi: 10.1371/journal.pone.0287361 (PMC10569536; doi:10.1371/journal.pone.0287361)
Supplement: Peer review history

# Peer review: Decision Letter 0

**DOI:** 10.1371/journal.pone.0287361.r001

**Authors:** Isabel Cristina Gonçalves Leite

14 Sep 2023

PONE-D-23-16973Factors related to the performance of the periodontal specialty in secondary oral health in Brazil.PLOS ONE

Dear Dr. Simião,

Thank you for submitting your manuscript to PLOS ONE. After careful consideration, we feel that it has merit but does not fully meet PLOS ONE’s publication criteria as it currently stands. Therefore, we invite you to submit a revised version of the manuscript that addresses the points raised during the review process.

Please submit your revised manuscript by Oct 29 2023 11:59PM. If you will need more time than this to complete your revisions, please reply to this message or contact the journal office at plosone@plos.org. When you're ready to submit your revision, log on to https://www.editorialmanager.com/pone/ and select the 'Submissions Needing Revision' folder to locate your manuscript file.

Please include the following items when submitting your revised manuscript:

- A rebuttal letter that responds to each point raised by the academic editor and reviewer(s). You should upload this letter as a separate file labeled 'Response to Reviewers'.
- A marked-up copy of your manuscript that highlights changes made to the original version. You should upload this as a separate file labeled 'Revised Manuscript with Track Changes'.
- An unmarked version of your revised paper without tracked changes. You should upload this as a separate file labeled 'Manuscript'.

If you would like to make changes to your financial disclosure, please include your updated statement in your cover letter. Guidelines for resubmitting your figure files are available below the reviewer comments at the end of this letter.

If applicable, we recommend that you deposit your laboratory protocols in protocols.io to enhance the reproducibility of your results. Protocols.io assigns your protocol its own identifier (DOI) so that it can be cited independently in the future. For instructions see: https://journals.plos.org/plosone/s/submission-guidelines#loc-laboratory-protocols. Additionally, PLOS ONE offers an option for publishing peer-reviewed Lab Protocol articles, which describe protocols hosted on protocols.io. Read more information on sharing protocols at https://plos.org/protocols?utm_medium=editorial-email&utm_source=authorletters&utm_campaign=protocols.

We look forward to receiving your revised manuscript.

Kind regards,

Isabel Cristina Gonçalves Leite

Academic Editor

PLOS ONE

Journal requirements:

When submitting your revision, we need you to address these additional requirements.

1. Please ensure that your manuscript meets PLOS ONE's style requirements, including those for file naming. The PLOS ONE style templates can be found at

https://journals.plos.org/plosone/s/file?id=wjVg/PLOSOne_formatting_sample_main_body.pdf and

https://journals.plos.org/plosone/s/file?id=ba62/PLOSOne_formatting_sample_title_authors_affiliations.pdf

Reviewer's Responses to Questions

**Comments to the Author**

1. Is the manuscript technically sound, and do the data support the conclusions?

The manuscript must describe a technically sound piece of scientific research with data that supports the conclusions. Experiments must have been conducted rigorously, with appropriate controls, replication, and sample sizes. The conclusions must be drawn appropriately based on the data presented. 

Reviewer #1: Partly

Reviewer #2: Yes

Reviewer #3: Yes

**********

2. Has the statistical analysis been performed appropriately and rigorously? 

Reviewer #1: I Don't Know

Reviewer #2: Yes

Reviewer #3: Yes

**********

3. Have the authors made all data underlying the findings in their manuscript fully available?

The PLOS Data policy requires authors to make all data underlying the findings described in their manuscript fully available without restriction, with rare exception (please refer to the Data Availability Statement in the manuscript PDF file). The data should be provided as part of the manuscript or its supporting information, or deposited to a public repository. For example, in addition to summary statistics, the data points behind means, medians and variance measures should be available. If there are restrictions on publicly sharing data—e.g. participant privacy or use of data from a third party—those must be specified.

Reviewer #1: Yes

Reviewer #2: Yes

Reviewer #3: Yes

**********

4. Is the manuscript presented in an intelligible fashion and written in standard English?

PLOS ONE does not copyedit accepted manuscripts, so the language in submitted articles must be clear, correct, and unambiguous. Any typographical or grammatical errors should be corrected at revision, so please note any specific errors here.

Reviewer #1: Yes

Reviewer #2: Yes

Reviewer #3: Yes

**********

5. Review Comments to the Author

Please use the space provided to explain your answers to the questions above. You may also include additional comments for the author, including concerns about dual publication, research ethics, or publication ethics. (Please upload your review as an attachment if it exceeds 20,000 characters)

Reviewer #1: Basically, the paper should be understandable just by reading the title, abstract, and tables. In that regard, the following points should be corrected first.

The use of the word secondary in the title needs to be clarified. If authors mean secondary analysis, I suggest using something like "Using DATASUS". Please clarify the definition of goal in outcome and suggest whether BDSC is not a more understandable acronym than CEO.

chart 1

Please write the PHC full name.

sum of periodontics workload is the number of visits?

CEO scope, CEO type. These terms alone do not convey understanding to the reader. The same goes for these expressions, Type 1,2,3.

Table 1

It’s undesirable to write ‘study outcome’ as the table title. I recommend to use the name of the specific outcome variables. Make it clear what the goal is, and if it's long, put the meaning in a footnote to the table.

Typically, the first table shows the results of a univariate analysis of how counfounders differ by dividing the outcome variable into columns before regression analysis. The second table shows the results of the regression, and the third table shows the results stratified by age, gender, or other important variables. In this study, there is only one table with the results, and why is there no demographic information such as age or gender?

Reviewer #2: Further analysis is needed in the future to analyze and verify the results after implementing the author's recommendations. The literature is needed to highlight the importance of periodontal health in developing countries.

Reviewer #3: The aim of the study is well written and the objectives defined .The methodology and the statistical analysis are well described and appropriate. As the authors have described in their study, the data maybe used as guidelines for better planning and implementation of specific actions and improving health policies for the public. Improvement in health policies and access to oral care is important to address a prevalence of periodontal disease as high as 34.4%to 63.8%.

**********

6. PLOS authors have the option to publish the peer review history of their article (what does this mean?). If published, this will include your full peer review and any attached files.

If you choose “no”, your identity will remain anonymous but your review may still be made public.

**Do you want your identity to be public for this peer review?** For information about this choice, including consent withdrawal, please see our Privacy Policy.

Reviewer #1: No

Reviewer #2: No

Reviewer #3: No

**********

# Author comment: Author response to Decision Letter 0

**DOI:** 10.1371/journal.pone.0287361.r002

26 Sep 2023

Reviewer #1

Basically, the paper should be understandable just by reading the title, abstract, and tables. In that regard, the following points should be corrected first.

Our response: We thank the reviewer for the positive feedback. We have revised the paper in response to the constructive and insightful reviewer’s comments.

The use of the word secondary in the title needs to be clarified. If authors mean secondary analysis, I suggest using something like "Using DATASUS". Please clarify the definition of goal in outcome and suggest whether BDSC is not a more understandable acronym than CEO.

Our response: We appreciate the suggestion. We have changed the title of the article and made other modifications to ensure greater textual clarity. 

Chart 1

Please write the PHC full name.

sum of periodontics workload is the number of visits?

CEO scope, CEO type. These terms alone do not convey understanding to the reader. The same goes for these expressions, Type 1,2,3.

Our response: We created a new column with the name 'Description of the variable' and provided descriptions for all variables to ensure the chart 1 is self-explanatory.

Table 1

It’s undesirable to write ‘study outcome’ as the table title. I recommend to use the name of the specific outcome variables. Make it clear what the goal is, and if it's long, put the meaning in a footnote to the table.

Our response: We have rewritten the title of Table 1 to address the reviewer's suggestion.

Typically, the first table shows the results of a univariate analysis of how counfounders differ by dividing the outcome variable into columns before regression analysis. The second table shows the results of the regression, and the third table shows the results stratified by age, gender, or other important variables. In this study, there is only one table with the results, and why is there no demographic information such as age or gender?

Our response: Dear reviewer, we understand your comment. However, in our article, the 1st level variables represent the team (institutional level), so variables related to gender and age do not apply in this context.

Reviewer #2 

Further analysis is needed in the future to analyze and verify the results after implementing the author's recommendations. The literature is needed to highlight the importance of periodontal health in developing countries.

Our response: We appreciate the reviewer's suggestion. We included a paragraph at the end of the Discussion section to emphasize the significance of periodontal health in developing countries.

Reviewer #3 

The aim of the study is well written and the objectives defined. The methodology and the statistical analysis are well described and appropriate. As the authors have described in their study, the data maybe used as guidelines for better planning and implementation of specific actions and improving health policies for the public. Improvement in health policies and access to oral care is important to address a prevalence of periodontal disease as high as 34.4%to 63.8%.

Our response: We thank the reviewer for the positive feedback. We included a paragraph at the end of the Discussion section emphasizing the importance of further studies in light of the increasing incidence of periodontal diseases.

Attachment Submitted filename: Response to reviewers.docx Click here for additional data file.

# Editor report: Decision Letter 1

**DOI:** 10.1371/journal.pone.0287361.r003

**Authors:** Isabel Cristina Gonçalves Leite

28 Sep 2023

Factors Affecting the Performance of Periodontal Specialty in Secondary Oral Health Care in Brazil

PONE-D-23-16973R1

Dear Dr. Simião,

We’re pleased to inform you that your manuscript has been judged scientifically suitable for publication and will be formally accepted for publication once it meets all outstanding technical requirements.

Within one week, you’ll receive an e-mail detailing the required amendments. When these have been addressed, you’ll receive a formal acceptance letter and your manuscript will be scheduled for publication.

An invoice for payment will follow shortly after the formal acceptance. To ensure an efficient process, please log into Editorial Manager at http://www.editorialmanager.com/pone/, click the 'Update My Information' link at the top of the page, and double check that your user information is up-to-date. If you have any billing related questions, please contact our Author Billing department directly at authorbilling@plos.org.

If your institution or institutions have a press office, please notify them about your upcoming paper to help maximize its impact. If they’ll be preparing press materials, please inform our press team as soon as possible -- no later than 48 hours after receiving the formal acceptance. Your manuscript will remain under strict press embargo until 2 pm Eastern Time on the date of publication. For more information, please contact onepress@plos.org.

Kind regards,

Isabel Cristina Gonçalves Leite

Academic Editor

PLOS ONE

Additional Editor Comments:

In view of the corrections made by the authors, and the relevance of the topic on secondary care in Dentistry, I consider that the article meets the conditions for publication.

# Editor report: Acceptance letter

**DOI:** 10.1371/journal.pone.0287361.r004

**Authors:** Isabel Cristina Gonçalves Leite

3 Oct 2023

PONE-D-23-16973R1 

Factors Affecting the Performance of Periodontal Specialty in Secondary Oral Health Care in Brazil 

Dear Dr. Simião:

I'm pleased to inform you that your manuscript has been deemed suitable for publication in PLOS ONE. Congratulations! Your manuscript is now with our production department. 

If your institution or institutions have a press office, please let them know about your upcoming paper now to help maximize its impact. If they'll be preparing press materials, please inform our press team within the next 48 hours. Your manuscript will remain under strict press embargo until 2 pm Eastern Time on the date of publication. For more information please contact onepress@plos.org.

If we can help with anything else, please email us at plosone@plos.org. 

Thank you for submitting your work to PLOS ONE and supporting open access. 

Kind regards, 

PLOS ONE Editorial Office Staff

on behalf of

Dr. Isabel Cristina Gonçalves Leite 

Academic Editor

PLOS ONE
